# Stability Criterion for the Assembly of Core–Shell Lipid–Polymer–Nucleic Acid Nanoparticles

**DOI:** 10.1021/acsnano.3c07204

**Published:** 2023-08-15

**Authors:** Juan L. Paris, Ricardo Gaspar, Filipe Coelho, Pieter A. A. De Beule, Bruno F. B. Silva

**Affiliations:** International Iberian Nanotechnology Laboratory, Braga, 4715-330, Portugal

**Keywords:** lipopolyplex, lipid−polycation−DNA (LPD), LPNP, membrane-coated nanoparticles, gene delivery, fluorescence cross-correlation spectroscopy (FCCS)

## Abstract

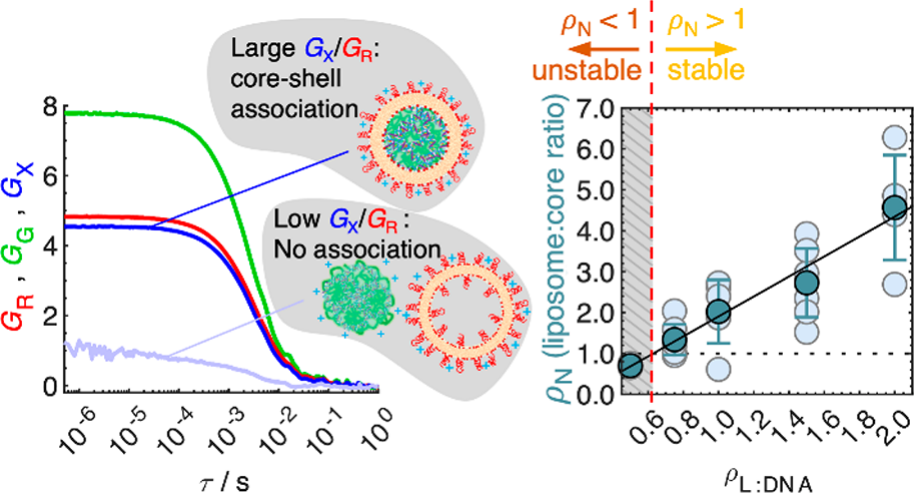

Hybrid core–shell lipid–polycation–nucleic acid nanoparticles (LPNPs) provide unique delivery strategies for nonviral gene therapeutics. Since LPNPs consist of multiple components, involving different pairwise interactions between them, they are challenging to characterize and understand. Here, we propose a method based on fluorescence cross-correlation spectroscopy to elucidate the association between the three LPNP components. Through this lens, we demonstrate that cationic lipid shells (liposomes) do not displace polycations or DNA from the polycation–DNA cores (polyplexes). Hence, polyplexes and liposomes must be oppositely charged to associate into LPNPs. Furthermore, we identify the liposome:polyplex number ratio (*ρ*_*N*_), which was hitherto an intangible quantity, as the primary parameter predicting stable LPNPs. We establish that *ρ*_*N*_ ≥ 1 ensures that every polyplex is enveloped by a liposome, thus avoiding coexisting oppositely charged species prone to aggregation.

## Introduction

Nonviral gene therapeutics to treat genetic and acquired diseases have become clinical reality through the introduction of an siRNA-based treatment for hereditary transthyretin-mediated amyloidosis^1^ and lipid-based mRNA vaccines for COVID-19.^2^ Further applications in the pipeline include personalized cancer vaccines^3,4^ and permanent knockout of defective genes using CRISPR/Cas9.^5^ Most nonviral gene formulations reaching the clinic rely on the use of ionizable cationic lipid formulations as nanocarriers. These materials readily associate with the oppositely charged nucleic acids (NAs), forming nanoassemblies that can be therapeutically efficient *in vivo*.^1,6−9^ Notwithstanding this success, the efficacy of nonviral gene therapeutics remains limited, particularly for therapeutic targets beyond the liver.^10,11^

**Figure 1:**
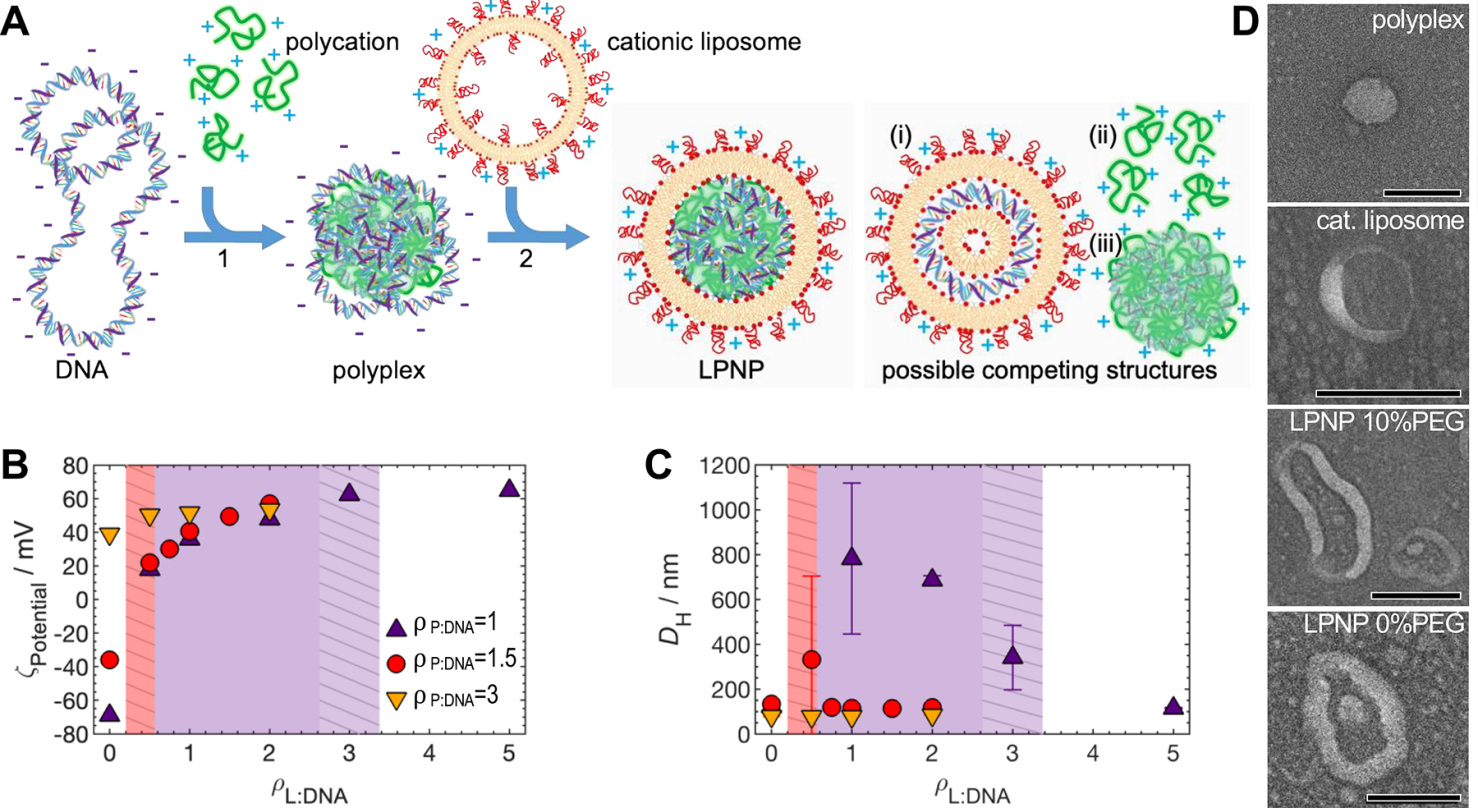
**Hybrid lipid–polymer nanoparticle (LPNP) assembly and physical properties.** (**A**) Schematic of LPNP assembly, consisting of a preliminary step (1) where DNA and polycations are mixed to form polyplexes with excess negative charge and a second step (2) where polyplexes are mixed with cationic liposomes to form LPNPs. Along with (or instead of) LPNP formation, hypothetical competing structures can form, such as (i) lipid–DNA complexes, (ii) polycations released from the polyplex core, or (iii) positively charged polyplexes stripped from the outer DNA layers. (**B**) *ξ*_Potential_ and (**C**) hydrodynamic diameter (*D*_H_) of the three LPNP systems as a function of *ρ*_L__:DNA_. The red dashed area represents the region of the *ρ*_P__:DNA_ = 1.5 system where aggregation was observed in some samples. The purple area represents the region of the *ρ*_P__:DNA_ = 1 system where large aggregation was observed, and the purple dashed area represents the region where aggregation was observed only in some samples. Data are means ± SD (*N* = 3). (**D**) TEM images of polyplexes, liposomes, and LPNPs with 10 and 0 mol % PEG. The scale bar is 100 nm.

Hybrid lipid–polycation–nucleic acid nanoparticles (LPNPs),^12−19^ which consist of a core composed by polycations complexed with NAs (i.e., polyplexes^20^), enveloped by a lipid membrane (Figure 1A), are promising delivery materials for next generation nonviral gene therapeutics. Since the polyplex core and lipid shell components can be independently designed, functionality can be improved to overcome the many physiological barriers to delivery.^13,14,21^ LPNPs are, however, complex systems. The three ionic components imply different pairwise electrostatic interactions between them (polycation–DNA, liposome–DNA, and liposome–polycation), which may dictate alternative structures depending on their relative strengths and method of assembly.^22,23^ Namely, cationic membranes may interact more favorably with NAs than polycations, excluding the latter from the assembly.^22^ This may easily be missed by most common characterization approaches, like dynamic light scattering (DLS) and electrophoretic mobility, which provide no direct evidence for formation of LPNPs comprising all three components. Hence, to this date, a suitable predictive model for LPNP formation is still missing, making the assembly of LPNPs mostly empiric, and limiting their potential.

Here we propose an approach based on dual-color fluorescence cross-correlation spectroscopy (FCCS)^24−26^ to quantify and elucidate the association between polyplexes and liposomes to form LPNPs. With this information, a picture of LPNP assembly is obtained, which could allow for improved efficiency gene therapeutics. We foresee that the methodology and insights shown here will also impact the understanding and development of other self-assembled composite nanosystems, including the promising cell-membrane-coated (camouflaged) nanoparticles.^27,28^

## Results and Discussion

We assemble core–shell LPNPs in two steps (Figure 1A). The polyplex core is assembled first by mixing polylysine (a common polycation in both polyplex and LPNP systems^20,29^) with DNA at polycation–amine to DNA–phosphate molar ratios (*ρ*_P__:DNA_) of 1, 1.5, and 3. *ρ*_P__:DNA_ = 1 polyplexes are negatively overcharged with DNA, showing negative *ξ*_Potential_ (Figure 1B), and coexist with a significant fraction of non-complexed DNA.^29^*ρ*_P__:DNA_ = 1.5 polyplexes are close to the polylysine–DNA isoelectric point (*ρ*_P:DNA__.iso_ = 1.6), showing a smaller negative *ξ*_Potential_ and virtually no free DNA or polylysine. Conversely, *ρ*_P__:DNA_ = 3 polyplexes are cationic, coexisting with excess polylysine. All the starting polyplexes and liposomes have hydrodynamic diameters (*D*_H_) below 140 nm (Figure 1C).

In the second step, liposomes composed of 80 mol % (molar percentage) cationic lipid and varying amounts of PEG-lipid are added to form core–shell LPNPs at various cationic lipid to DNA phosphate molar ratios (*ρ*_L__:DNA_). Parts B and C of Figure 1 indicate two trends for LPNPs, depending on the *ξ*_Potential_ of the starting polyplex. For cationic polyplexes (*ρ*_P__:DNA_ = 3), neither *D*_H_ nor *ξ*_Potential_ are significantly affected when cationic liposomes are added. Conversely, when the starting polyplexes are anionic (*ρ*_P__:DNA_ = 1 and 1.5), the LPNP charge switches to cationic already at the lowest *ρ*_L__:DNA_ additions, coinciding with the observation of strong aggregation (*D*_H_ > 500 nm), especially for *ρ*_P__:DNA_ = 1. Further addition of liposomes leads to stabilization. Clear solutions with *D*_H_ < 120 nm are obtained for *ρ*_L__:DNA_ = 5 (for *ρ*_P__:DNA_ = 1) and *ρ*_L__:DNA_ = 0.75 (for *ρ*_P__:DNA_ = 1.5) LPNPs. Since *ρ*_P__:DNA_ = 1 polyplexes are more anionic and coexist with free DNA (which needs to be neutralized), more liposomes are needed to stabilize these LPNPs than the *ρ*_P__:DNA_ = 1.5 ones. However, at first, the factor of ca. 6 times larger *ρ*_L__:DNA_ needed to stabilize *ρ*_P__:DNA_ = 1 polyplexes seems high.

Negative polyplexes aggregating initially and then redispersing for increasing *ρ*_L__:DNA_ hint at strong electrostatic interactions with liposomes and conversion into LPNPs. TEM results (Figure 1D) also suggest formation of LPNPs, with imaged particles showing a core like the polyplex samples, enveloped by a membrane. However, one should also consider other interactions potentially resulting in structures competing with LPNPs. For instance, DNA could be partially or entirely displaced from the polycation to the cationic liposomes to form liposome–DNA complexes (Figure 1A), similarly to what happens when histone–DNA nucleosomes are mixed with cationic liposomes.^22^ Such structures could have sizes and *ξ*_Potential_ like LPNPs. Regarding the cationic polyplexes (*ρ*_P__:DNA_ = 3), the lack of observable changes with *ρ*_L__:DNA_ hints at no formation of LPNPs, but also here one should consider the possibility of cationic liposomes displacing some polycations to form LPNPs. These alternative scenarios are difficult to discern with DLS/electrophoretic mobility and even with TEM.

FCCS can overcome this limitation by providing a quantitative measure of co-localization between two fluorescently labeled species (along with their sizes).^24−26,30−32^ We labeled polylysine covalently with Atto-488 (green) and likewise the cationic liposomes with Texas Red. In the *ρ*_P__:DNA_ = 3 system, DNA is labeled with YOYO-1 (green) instead of polylysine, to avoid the complication of having labeled non-complexed polylysine in solution. Single channel FCS auto-correlation curves for the different polyplex and liposome starting formulations can be found in Figure S1.

**Figure 2:**
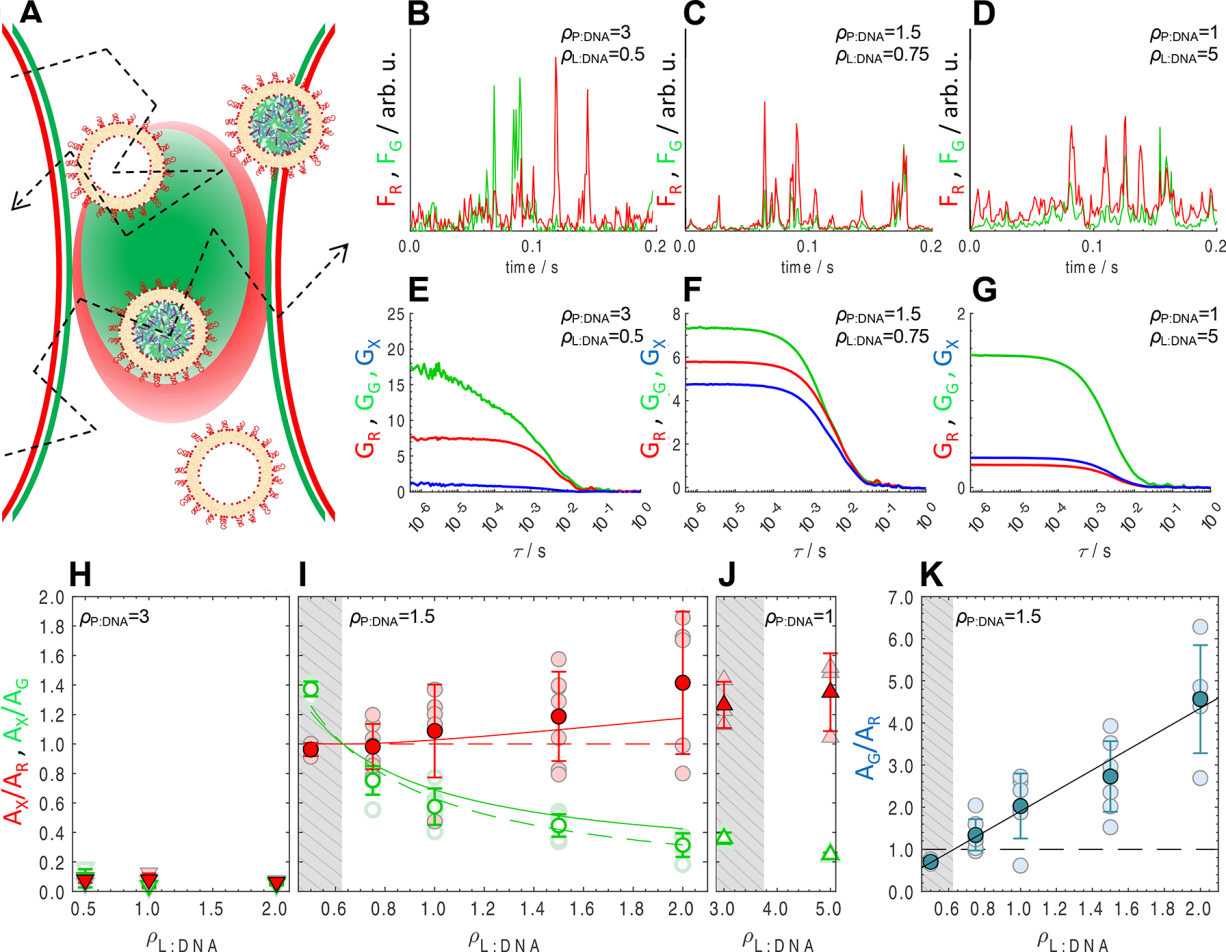
**Quantification of association between PEGylated cationic liposomes and polyplexes with FCCS.** (**A**) Schematic of the green and red confocal volumes (in green and red, respectively) with LPNPs and cationic liposomes diffusing through them. The dashed line represents a random walk of a particle diffusing through the overlapping confocal volumes. (**B**–**D**) Representative fluorescence time traces for samples *ρ*_P__:DNA_ = 3, *ρ*_L__:DNA_ = 0.5; *ρ*_P__:DNA_ = 1.5, *ρ*_L__:DNA_ = 0.75; and *ρ*_P__:DNA_ = 1, *ρ*_L__:DNA_ = 5, respectively. (**E**–**G**) The corresponding auto- and cross-correlation curves for the same samples. The green and red fluorescence (*F*_G_ and *F*_R_) and auto-correlation curves (*G*_G_ and *G*_R_) are shown in green and red, respectively. The cross-correlation curves (*G*_X_) are shown in blue. (**H**–**J**) The  (red filled symbols) and  (green open symbols) ratios provide the fraction of polyplexes converted to LPNPs (*f*_LPNP_) and the fraction of liposomes used in LPNPs, respectively (eq 4a). Individual measurements are represented by light-colored symbols, while their means and respective error bars are represented in darker colors. The green and red dashed lines in (I) show the  and  expressions, expected for a 1:1 stoichiometry (no fitting parameters). The green and red straight lines show the 1:*n* stoichiometry-modified  and  expressions (eq S11, section S3), both fitted simultaneously to the data. (**K**) The  ratio (for *ρ*_P__:DNA_ = 1.5), which is equivalent to the liposome:polyplex ratio (*ρ*_N_). The boundary between marginal and robust stability (*ρ*_N_ = 1) is estimated at *ρ*_L__:DNA_ = 0.63 for *ρ*_P__:DNA_ = 1.5. The gray-dashed areas in (H–K) delineate the compositions of marginal stability, in which some samples were not measurable due to the presence of aggregates. The liposome PEGylation degree in all data is 10 mol %.

In FCCS, the confocal volumes defined by the green and red lasers of a confocal microscope are fixed in space, while labeled species diffuse through the overlapping green and red region (Figure 2A). If polyplexes and liposomes do not form LPNPs, they diffuse independently, producing uncorrelated spikes in both green and red fluorescence signals (*F*_G_ and *F*_R_, respectively). This is the case for the *ρ*_P__:DNA_ = 3 system, in which polyplexes and cationic liposomes show unsynchronized spikes in both channels (Figure 2B). In contrast, if polyplexes and liposomes form LPNPs, they diffuse together producing correlated spikes. This is the case for the *ρ*_P__:DNA_ = 1.5 system (Figure 2C), where excellent overlap between the green and red signals is observed. Since here both labeled species are cationic (polylysine and liposomes), their co-localization indicates strong polyplex–liposome association. In the *ρ*_P__:DNA_ = 1 case (Figure 2D), the spikes in the green channel overlap with red spikes, but there are many red spikes that have no overlap with green. This suggests coexistence of LPNPs with excess liposomes or liposome–DNA complexes, which would produce only red spikes. Overall, these observations confirm LPNP formation when the charge is being inverted (*ρ*_P__:DNA_ = 1 and 1.5) but not when the charge of polyplexes and liposomes has the same sign (*ρ*_P__:DNA_ = 3).

For a quantitative analysis, the auto- and cross-correlation curves of the time-dependent fluorescence signals can be examined^24,26^ (Figure 2E–G). In the case of free diffusion in three dimensions, the correlation function *G* can be represented as follows:
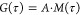
1The amplitude *A* in this equation contains information on the number of species in the confocal volume, while the lag-time (τ)-dependent part, *M*(τ), contains information on their dynamics:
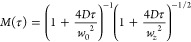
2Here, *w*_0_ and *w*_*z*_ are the lateral and axial radii of the detection volume, respectively, and *D* is the diffusion coefficient. By fitting eqs 1 and 2 to the experimental auto-correlation function, the diffusion time and amplitudes of the species can be determined. The Stokes–Einstein relation allows the calculation of the hydrodynamic diameter *D*_H_ through *D*, which is used to determine LPNP size (Table S4 and Figure S2). For a more comprehensive description of FCCS, readers are referred to the Supporting Information.

While the green and red auto-correlation curves (*G*_G_ and *G*_R_) describe the dynamics of species carrying the green and red labels, respectively, the cross-correlation curve (*G*_X_) results from the correlation between the two signals and therefore only contains information on the species carrying both labels. Most importantly, their amplitudes contain information on the number of each labeled species, and on the number of species containing both labels,^24^ according to
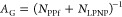
3a
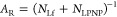
3b
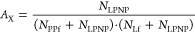
3cHere, *A*_G_ and *A*_R_ are the amplitudes of the green and red auto-correlation, respectively, and *A*_X_ is the amplitude of the cross-correlation. *N*_PPf_, *N*_Lf_, and *N*_LPNP_ are the average number of free polyplexes, free liposomes, and LPNPs in the confocal volumes, respectively. Notably, the ratio between *A*_X_ and *A*_R_ provides a direct measure of the fraction of polyplexes converted to LPNPs (*f*_LPNP_), according to
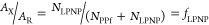
4aConversely, the  ratio provides a direct measure of the fraction of cationic liposomes used to make LPNPs:
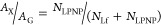
4bEquations 3 and 4 assume, as a simplification, one polyplex associating with one cationic liposome (1:1 stoichiometry) to form one LPNP.^24^ In sections S2 and S3 of the Supporting Information we detail why a much more complex model based on a 1:*n* stoichiometry with *n* liposomes per LPNP to estimate *f*_LPNP_ is generally not required.

Inspection of Figure 2E and H shows that the  ratio is very low, confirming that cationic polyplexes (*ρ*_P__:DNA_ = 3) practically do not associate with cationic liposomes to form LPNPs ().

In contrast, systems containing mildly anionic polyplexes (*ρ*_P__:DNA_ = 1.5) show much larger *A*_X_ values, comparable to both *A*_G_ and *A*_R_, indicating strong co-localization (Figure 2F). The  ratio, which is equivalent to *f*_LPNP_ (eq 4a), is shown to increase from ca. 1 to 1.42 as *ρ*_L__:DNA_ is raised from 0.5 to 2 (Figure 2I). This indicates practically full conversion of polyplexes into LPNPs (*f*_LPNP_ ≈ 1) in detriment of the possible competing structures depicted in Figure 1A. The fact that the average  values become greater than 1 for higher *ρ*_L__:DNA_ indicates that some LPNPs may have more than one liposome attached, although this deviation is only significant at the largest *ρ*_L__:DNA_ = 2, when many liposomes are added. We will return to this point later.

Figure 2I also shows the  ratio, which directly provides the fraction of liposomes used to make LPNPs (eq 4b). As *ρ*_L__:DNA_ is increased,  starts to steadily decrease, indicating that, after full LPNP conversion is reached, most additional liposomes are in excess, as expected for a 1:1 stoichiometry.

At this point of nearly complete conversion of polyplexes to LPNPs (*f*_LPNP_ ≈ 1), we can affirm that *N*_PPf_ = 0. This allows us to directly determine *N*_LPNP_ from *A*_G_^–1^ (eq 3a). With *N*_LPNP_ known, within the 1:1 stoichiometry approximation, *N*_Lf_ can be directly obtained through *A*_R_^–1^ (eq 3b).

We now focus our attention on the region with marginal stability (shaded area in Figure 2I). It is noticeable that  is greater than 1 for *ρ*_L__:DNA_ = 0.5, which similarly to the case of  > 1 indicating that some LPNPs may have more than one liposome attached, suggests that for this composition there may not be enough liposomes to completely coat all polyplexes, and there might be instances of two polyplexes sharing one liposome. This could explain the greater instability.

The ratio between the number of liposomes and polyplexes (*ρ*_*N*_) is not straightforward to obtain with most techniques but is easily accessible using FCCS. The total number of polyplexes and liposomes in solution is obtained through *A*_G_^–1^ and *A*_R_^–1^, respectively (eqs 3a and 3b), and hence, the  ratio provides *ρ*_*N*_.

Figure 2K shows that, for the *ρ*_P__:DNA_ = 1.5 system, all the samples in the stable region (*ρ*_L__:DNA_ ≥ 0.75) have a *ρ*_*N*_ ratio greater than 1. Conversely, the samples in the marginally stable region (*ρ*_L__:DNA_ = 0.5) show a *ρ*_*N*_ slightly below 1. This strongly hints that *ρ*_*N*_ ≥ 1 is a critical requirement for robust colloidal stability, suggesting that all polyplexes are coated by one liposome and converted to LPNPs. Conversely, *ρ*_*N*_ < 1 indicates that some polyplexes will be transformed into cationic LPNPs while some others will remain anionic, ultimately leading to aggregation.

However intuitive, this *ρ*_*N*_ ≥ 1 requirement is a critical insight. The reason why we and others have failed to notice *ρ*_*N*_ earlier likely results from its hitherto intangible nature. *ρ*_*N*_ is difficult to estimate beforehand and to determine experimentally. Furthermore, it depends on sample preparation. Hence, *ρ*_*N*_ is not salient and tends to be overlooked. FCCS then plays a crucial role in revealing and highlighting *ρ*_*N*_ by measuring both the number of polyplexes and liposomes simultaneously.

Since *ρ*_*N*_ = 1 is reached at *ρ*_L__:DNA_ = 0.63, for a 1:1 stoichiometry and *f*_LPNP_ = 1, the ratio between the total number of liposomes and polyplexes is given by *ρ*_L__:DNA_/0.63. Hence, the number of unused liposomes can be estimated by  and eq 4a rewritten as  and  (cf., section S3). Both expressions are plotted in Figure 2I. Without any adjustable parameters, the agreement with the data is remarkable for . Notably, the model also reproduces the  > 1 behavior observed for *ρ*_L__:DNA_ = 0.5, corroborating our interpretation that in this regime there are not enough liposomes to coat all polyplexes. For , the discrepancy between model and data is more noticeable at larger *ρ*_L__:DNA_, hinting that having many free liposomes results in a higher probability of some LPNPs being enveloped by an additional lipid bilayer. For a 1:*n* polyplex:liposome stoichiometry, this can be modeled by modifying the  and  expressions (eq S11) to include the term , where *p* is the probability of a free liposome enveloping an existing LPNP. The mutual best fit for both  and  produces *p* = 0.19 (Figure 2I). While the  line now fits the data better, the agreement of the  line worsens. Overall, this confirms that *f*_LPNP_ ≈ 1 and that the 1:1 approximation is generally suitable, although some deviations, partially captured by the 1:*n* model above, occur when *ρ*_*N*_ is large.

Regarding the *ρ*_P__:DNA_ = 1 system, the results are qualitatively similar to *ρ*_P__:DNA_ = 1.5 (Figure 2G and J). Namely, the  indicates that all polyplexes are converted into LPNPs (*f*_LPNP_ ≈ 1), and *n* is slightly greater than 1. Hence, even though these polyplexes are more charged, one liposome per polyplex is, on average, still sufficient to envelop and convert them into LPNPs. The much larger *ρ*_L__:DNA_ ratio needed to stabilize *ρ*_P:DNA_ = 1 polyplexes can then be explained, on one hand, by the initial excess free DNA coexisting with polyplexes,^29^ which requires additional liposomes to consume it (also explaining the much lower  ratio observed). On the other hand, as evident from *A*_G_^–1^ (Figure 2F,G and Table S5), the number of polyplexes is greater in the *ρ*_P__:DNA_ = 1 system by a factor of ca. 3–5 times (eq 3a). Thus, to ensure the *ρ*_*N*_ ≥ 1 requirement, the number of liposomes needed to stabilize the system also increases.

A coherent picture hence emerges describing LPNP assembly based solely on the initial polyplex charge, which needs to be opposite to the liposome charge for LPNPs to form, and on *ρ*_*N*_ ≥ 1. If polyplexes coexist with excess DNA, extra liposomes are needed to consume it, to avoid coexistence of species with opposite charge. In such cases, LPNPs coexist with liposome–DNA complexes. This elusively simple behavior results from the strong interaction between polylysine and DNA, in which none is displaced when cationic liposomes are added. The elucidation of LPNP assembly thus allows their formulation in a rational, controlled, and predictable manner, enabling reliable and systematic studies of LPNP formulations and the establishment of well-defined structure–efficiency relations. We foresee that this will have a significant impact on the transfection efficiency of therapeutic agents based on LPNPs.

**Figure 3:**
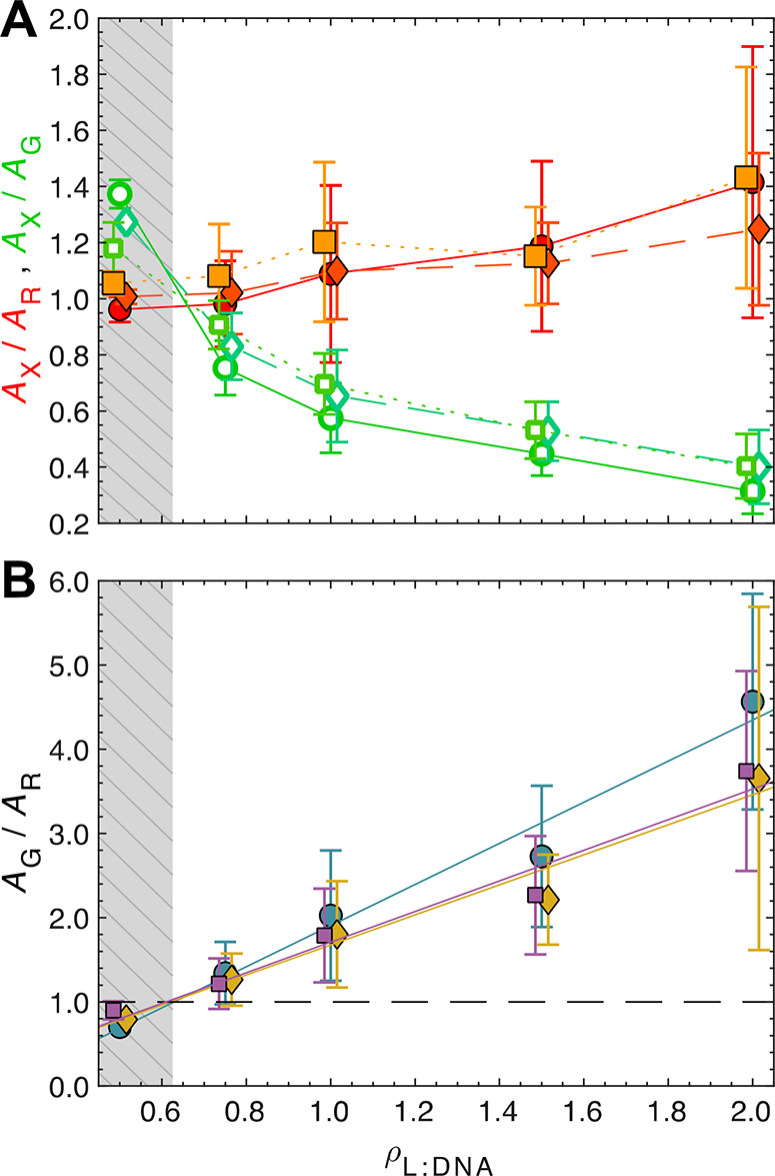
**Effect of liposome PEGylation in the assembly of LPNPs.** (**A**)  (red-orange symbols) and  (green-blue symbols) ratios for LPNP systems with 0 mol % PEG (squares), 5 mol % PEG (diamonds), and 10 mol % PEG (circles) liposomes. The trends are similar in the three systems, but there is a systematic small increase of the  and  pair for lower degrees of liposome PEGylation. (**B**)  ratio for LPNP systems with 0 mol % PEG (squares), 5 mol % PEG (diamonds), and 10 mol % PEG (circles) liposomes. The boundaries between marginal and robust stability (*ρ*_N_ = 1) are the same for the three systems (*ρ*_L__:DNA_ = 0.63). Data in (A) and (B) is offset by *ρ*_L__:DNA_ ± 0.015 for easier visualization. The gray-dashed areas delineate the compositions of marginal stability. Data are means ± SD (*N* ≥ 3).

The findings above are largely reproduced when the PEG% in liposomes is reduced from 10 to 5 and 0 mol % (Figure 3A). Most importantly, also here we find *ρ*_*N*_ ≥ 1 for all stable LPNPs and *ρ*_*N*_ < 1 for the nonstable ones (Figure 3B), reinforcing that *ρ*_*N*_ ≥ 1 is critical for LPNP stability. The main difference resides in a small increase of both  and  ratios when the PEG% in liposomes decreases. This indicates a small increase in *p* (cf., section S3, Figure S3, and Table S6), meaning that low-PEG liposomes associate more extensively with polyplexes (probably because PEG acts as a steric barrier against adsorption^1^).

We postulate that the general features of LPNP assembly observed here can be extended to other LPNP systems containing reasonably strong polycations and cationic liposomes with monovalent ionizable/cationic lipid membranes of similar rigidity. The experimental approach detailed here can also be extended to quantify the association between different components in diverse composite materials within and beyond gene therapeutics (e.g., cell-membrane-coated nanoparticles^27,28^), providing insights into their assembly, stability, and structure–function relations.

## Conclusions

In this work we have explored the association of polylysine–DNA polyplex cores with cationic liposome shells to form LPNPs using FCCS. With this technique, we have shown that, for polyplex cores and liposome shells to associate and form LPNPs, they must be oppositely charged. Importantly, we have also shown that the liposome:polyplex number ratio (*ρ*_*N*_), as opposed to the more common charge ratio, is a critical parameter to predict stable LPNP formation. We find that *ρ*_*N*_ ≥ 1 is required to ensure that every polyplex is enveloped by a liposome, thus avoiding the coexistence of oppositely charged species and inhibiting aggregation. Furthermore, we have observed significant differences in polyplex behavior, depending on the *ρ*_P__:DNA_ ratio. When further away from the isoelectric point, polyplexes are more numerous and coexist with free DNA, requiring more liposomes to produce LPNPs. Such particles are likely to contain smaller amounts of DNA and coexist with lipoplexes, which should be taken into consideration in formulation design. These findings were largely reproduced when varying the degree of liposome PEGylation, which further strengthens our observations about the assembly and stability of LPNP systems. Finally, since the chosen LPNP components are common, these observations and FCCS methodology should be applicable to many systems, including other self-assembly composite materials for applications beyond gene delivery.

## Experimental Section

### Materials

The lipids 1,2-dioleoyl-3-trimethylammonium-propane (DOTAP), 1,2-dioleoyl-*sn*-glycero-3-phosphocholine (DOPC), and 1,2-distearoyl-*sn*-glycero-3-phosphoethanolamine-*N*-[amino(polyethylene glycol)-2000] (DSPE-PEG) solubilized in chloroform were purchased from Avanti Polar Lipids and used as received. Poly-l-lysine solution 0.1% (w/v) in water was purchased from Sigma-Aldrich. GFP plasmid (pCMV-GFP), obtained through Addgene, was a gift from Connie Cepko (Addgene plasmid #11153).^33^ The dye Atto 488-NHS was purchased from ATTO-TEC GmbH. Texas Red 1,2-dihexadecanoyl-*sn*-glycero-3-phosphoethanolamine, triethylammonium salt (Texas Red-labeled lipid), and the dye YOYO-1 were purchased from ThermoFisher Scientific Inc. All the aqueous solutions were prepared with DNase-free and RNase-free Milli-Q water.

### Characterization Techniques

The different nanostructures obtained in this work were characterized by the following techniques: DLS and electrophoretic mobility (*ξ*_Potential_) were performed with a SZ-100 device from Horiba, measuring at a scattering angle of 173°. Transmission electron microscopy (TEM) of the samples after negative staining with UranyLess (following the manufacturer’s instructions) was performed with a JEOL 2100 200 kV TEM. FCCS was performed in a confocal microscope LSM780 from Zeiss following a previously described protocol.^24^ Each independent sample was measured with 40 repeats of 5 s each, with the final auto- and cross-correlation curves being averaged out. Typically, five-to-seven independent samples were prepared and measured with FCCS for each composition. The excitation laser lines used were 488 nm for Atto 488 dye and 561 nm for Texas Red. The laser power was set so that the intensity in the “red” (561 nm) channel was slightly above that in the “green” (488 nm) channel, thus minimizing overestimation of cross-correlation due to crosstalk.^24^ Besides minimizing crosstalk experimentally, the data is further corrected for crosstalk according to the procedure described in Bacia et al.^34^ The confocal volumes were calibrated using fluorophores with a known diffusion coefficient, and the overlap volume was determined by employing a mixture of single-labeled green (Atto 488) and red (Texas Red) liposomes and double-labeled liposomes with the same fluorescent labels (see section S4,Supporting Information). A related calibration approach was described recently by Werner et al.^35^ FCCS auto- and cross-correlation curves were fitted to a 3D normal diffusion model using QuickFit 3.0 software and home-built Matlab scripts.

### Labeling of Polylysine with Atto 488 Dye

Polylysine was labeled by reacting the polymer with Atto 488-NHS (ratio 1:100 Atto dye:NH_2_ groups in the polymer). After stirring in aqueous solution at room temperature overnight, the labeled polymer was obtained. The purification was performed by size exclusion chromatography using a Sephadex G25 stationary phase.

### Preparation of LPNPs

Polyplexes were prepared by mixing aqueous solutions of plasmidic DNA and polylysine at *ρ*_P__:DNA_ ratios (ratio between amino groups in the polymer to phosphate groups in the plasmid) of 1, 1.5, and 3 and incubating them at room temperature for 30 min in an orbital shaker.

PEGylated and non-PEGylated cationic liposomes with a lipid molar composition of DOTAP:DOPC:DSPE-PEG of 80:20-*x*:*x* (with *x* = 10, 5 and 0%) were prepared. First, the lipids were mixed in chloroform solution, followed by evaporation of the solvent, rehydration with water, and sonication with a tip sonicator at a final total lipid concentration of 4 mM (sonication conditions: 10% amplitude, 1 min, 50% duty cycle using a Branson Digital Sonifier 250 Model).

Finally, both components (polyplex and liposomes) were mixed at different proportions, with a lipid amine to DNA phosphate ratio (*ρ*_L__:DNA_) in the range of 0.5–5. A final polylysine concentration of 7 μg/mL was fixed for all the prepared samples. The obtained dispersions were characterized by *ξ*_Potential_ and DLS.

For FCCS, the LPNPs were prepared following the same protocol but employing labeled components. Atto 488-labeled polylysine was used to prepare the polyplexes, while Texas Red-labeled phospholipid was included in the liposomal formulation (0.2% total lipid weight). Samples with cationic polyplexes (*ρ*_P__:DNA_ = 3) were also prepared with YOYO 1-labeled DNA and Texas Red-labeled liposomes.

## Supporting Information Available

A detailed FCCS implementation description, 1:*n* polyplex:liposome stoichiometry model of LPNP formation, and determination of the overlap confocal volume; Figures S1–S5 and Tables S1–S6 are also provided (PDF)

## Notes

A preprint version of the work described in this article can be found at: Paris, J. L.; Gaspar, R.; Coelho, F.; De Beule, P. A. A.; Silva, B. F. B. Stability Criterion for the Assembly of Hybrid Lipid-Polymer-Nucleic Acid Nanoparticles. 2022, doi: 10.1101/2022.02.06.479316. bioRxiv. https://www.biorxiv.org/content/10.1101/2022.02.06.479316v2 (accessed August 9, 2023).
