# Supplementary material for: Stability Criterion for the Assembly of Core–Shell Lipid–Polymer–Nucleic Acid Nanoparticles
Source: ACS Nano. 2023 Aug 15;17(17):17587–94. doi: 10.1021/acsnano.3c07204 (PMC10510699; doi:10.1021/acsnano.3c07204)
Supplement: Supplementary file 1 — nn3c07204_si_001.pdf [file nn3c07204_si_001.pdf]

SUPPORTING INFORMATION

for

Stability Criterion for the Assembly of Core-Shell Lipid-Polymer-Nucleic Acid Nanoparticles

Juan L. Paris[1*#], Ricardo Gaspar[1], Filipe Coelho[1], Pieter A. A. De Beule[1], Bruno F. B. Silva[1,3*‡]

[1]International Iberian Nanotechnology Laboratory; Braga, 4715-330, Portugal.

[#]Current address: Instituto de Investigación Biomédica de Málaga y Plataforma en Nanomedicina-IBIMA Plataforma BIONAND. Málaga, 29590, Spain.

[‡]Current address: Empa, Swiss Federal Laboratories for Materials Science and Technology, Center for X-ray Analytics, Laboratory for Biointerfaces, and Laboratory for Biomimetic Membranes and Textiles, CH-9014 St. Gallen, Switzerland.

*Corresponding authors. Email: juan.paris@ibima.eu, bruno.silva@empa.ch

Supplementary Text

S1. Fluorescence Cross-Correlation Spectroscopy background

In the most common experimental implementation of Fluorescence Cross-Correlation Spectroscopy (FCCS), two components of a system are labelled with two spectrally resolved fluorophores (*a* and *b*, typically emitting in the green and red spectra) and their fluorescence signals (F_a and F_b) are followed as they diffuse in and out of the two corresponding confocal volumes. The overlap between the two confocal volumes should be as large as possible. The dynamics of the components cause fluorescence intensity fluctuations that can be represented through the normalized correlation function (*G*), determined from the time-dependent fluorescent signals:

$$G_{ab}(\tau) = \frac{\left\langle \delta F_a(t)\, \delta F_b(t+\tau) \right\rangle}{\left\langle F_a(t) \right\rangle \left\langle F_b(t) \right\rangle} \tag{S1}$$

Here $\delta F(t) = F(t) - \left\langle F(t) \right\rangle$ and τ is the lag time. Autocorrelation functions use only one signal (*a*=*b*) and represent the dynamics of species containing that signal. The cross-correlation uses both signals (*a*≠*b*) and represents only the dynamics of species containing the two labelled components. FCCS can therefore be used to quantify colocalization between two labelled species. For simplicity the cross-correlation function will be denoted by G_X.

In the case of free diffusion in three dimensions, the correlation function *G* can be represented through:

$$G(\tau) = A \cdot M(\tau) \tag{S2}$$

where the amplitude *A* contains information on the number of species, and $M(\tau)$, which contains information regarding their dynamics, is given by:

$$M(\tau) = \left(1+\frac{4D\tau}{w_0^2}\right)^{-1}\left(1+\frac{4D\tau}{w_z^2}\right)^{-1/2} = \left(1+\frac{\tau}{\tau_D}\right)^{-1}\left(1+\frac{\tau}{\tau_D S^2}\right)^{-1/2} \quad \text{(S3)}$$

Here, $S = w_z / w_0$ is the aspect ratio between the axial and lateral radii of the detection volume (w_z and w_0, respectively), $\tau_D = w_0^2 / 4D$ is the diffusion time, and D is the diffusion coefficient. By fitting Eqs. S2-S3 to the experimental autocorrelation function, the diffusion time and amplitudes of the species are obtained. Through D, the hydrodynamic diameter D_H can be obtained through the Stokes-Einstein relation.

Figure S2 shows a comparison between sizes obtained with DLS and FCCS. FCCS size data has larger error bars since measurements are quasi-single particle, requiring long measurements or multiple measurements to obtain statistical results identical to DLS. Conversely, DLS measurements are more susceptible to bias towards larger averages when samples are polydisperse. The very good agreement between FCCS and DLS results indicates that LPNP samples have low polydispersity[1].

The relation between the amplitude of the autocorrelation function of signal *a* and the number of species contributing to it is given by:

$$A_a = \sum_i \eta_{i,a}^2 \cdot N_i \Big/ \left(\sum_i \eta_{i,a} \cdot N_i\right)^2 \quad \text{(S4a)}$$

Here N_i represents the average number of species *i* in the confocal volume of signal *a*, and $\eta_{i,a}$ represents the brightness of specie *i*, also on the fluorescent signal *a*. Conversely, the cross-correlation amplitude between signals *a* and *b*, A_X, is given by:

$$A_X = \sum_i \eta_{i,a} \cdot \eta_{i,b} \cdot N_i \Big/ \left[\left(\sum_i \eta_{i,a} \cdot N_i\right)\left(\sum_i \eta_{i,b} \cdot N_i\right)\right] \quad \text{(S4b)}$$

A_X therefore, provides quantitative information regarding the association between the species producing signals *a* and *b*.

S2. Implementing Fluorescence Cross-Correlation Spectroscopy to quantify formation of DNA-carrying hybrid lipid-polymer nanoparticles

In the present work, polylysine-DNA polyplexes (PPs) are combined with cationic liposomes (Ls) to form hybrid lipid-polymer-DNA nanoparticles (LPNPs). For the simplified case of: (i) one polyplex associating with one cationic liposome (1:1 stoichiometry) to form one LPNP; (ii) no crosstalk between the two channels; and (iii) no changes in the brightness of the species following the association, the equations relating the amplitudes of the correlation curves with the number of species in the confocal volume become straightforward (Eqs. 3a-c in the main text):

$$A_G = \left(N_{PPf} + N_{LPNP}\right)^{-1} \quad \text{(3a)}$$

$$A_R = \left(N_{Lf} + N_{LPNP}\right)^{-1} \quad \text{(3b)}$$

$$A_X = \frac{N_{LPNP}}{\left(N_{PPf} + N_{LPNP}\right) \cdot \left(N_{Lf} + N_{LPNP}\right)} \quad \text{(3c)}$$

Here A_G and A_R are the amplitudes of the green and red auto-correlation, respectively, and A_X is the amplitude of the cross-correlation. N_{PPf}, N_{Lf}, and N_{LPNP}, are the average number of free polyplexes, free liposomes and LPNPs in the confocal volumes, respectively.

For the more general case in which one polyplex associates with n liposomes to form a LPNP nanoparticle containing n liposomes, and, as before, assuming no crosstalk between the two channels

and no changes in the brightness of the species following the association, the equations relating the amplitudes of the correlation curves with the number of species in the confocal volume become:

$$A_G = \left(N_{PPf} + N_{LPNP}\right)^{-1} \tag{S5a}$$

$$A_R = \frac{\left(N_{Lf} + n^2 \cdot N_{LPNP}\right)}{\left(N_{Lf} + n \cdot N_{LPNP}\right)^2} \tag{S5b}$$

$$A_X = \frac{n \cdot N_{LPNP}}{\left(N_{PPf} + N_{LPNP}\right) \cdot \left(N_{Lf} + n \cdot N_{LPNP}\right)} \tag{S5c}$$

The A_X / A_R and A_X / A_G ratios are now given by:

$$\frac{A_X}{A_R} = f_{LPNP} \frac{n \cdot \left(N_{Lf} + n \cdot N_{LPNP}\right)}{\left(N_{Lf} + n^2 \cdot N_{LPNP}\right)} \tag{S6a}$$

and

$$\frac{A_X}{A_G} = \frac{n \cdot N_{LPNP}}{N_{Lf} + n \cdot N_{LPNP}} \tag{S6b}$$

where $f_{LPNP} = N_{LPNP} / \left(N_{PPf} + N_{LPNP}\right)$ (Eq. 4a). Note that for $n = 1$, Eq. S6 reduces to Eq. 4. Regardless of the value of n, the fraction of liposomes that associate with polyplexes continues to be given by the A_X / A_G ratio, but f_{LPNP} will no longer be given simply by A_X / A_R if $n > 1$, and depends now also on n and N_{Lf}. The latter term can be removed by combining Eq. S6b with Eq. S6a, and the fraction of coated LPNPs, f_{LPNP}, can now be obtained by:

$$f_{LPNP} = \frac{A_X^2 \cdot (n - 1) + A_X \cdot A_G}{n \cdot A_R \cdot A_G} \tag{S7}$$

Because both f_{LPNP} and n are unknowns, Eq. S7 still cannot be used alone to determine the fraction of coated L_nPNPs without knowledge of n. However, it can still be used to estimate expected A_X / A_R ratios for different scenarios (i.e. for different fractions of coated LPNPs and different n) and, by comparison with the experimentally observed A_X / A_R ratios, determine which scenarios are more likely.

S3. Fitting the experimental data with a $1{:}n$ polyplex:liposome stoichiometry model

Since the condition for stability $\rho_N = 1$ is reached at $\rho_{L:DNA.stab} = 0.63$ (Fig. 2K), the number of free liposomes can be estimated from the following expression, valid for $f_{LPNP} = 1$ and $\rho_N \geq 1$:

$$N_{Lf} = N_{LPNP}\left(\rho_{L:DNA} / \rho_{L:DNA.stab} - n\right) \tag{S8}$$

Here $\rho_{L:DNA.stab}$ is defined as the value of $\rho_{L:DNA}$ at which the $\rho_N = 1$ condition is met. In this work, for $\rho_{P:DNA} = 1.5$, $\rho_{L:DNA.stab}$ is fixed at 0.63, as determined in Fig. 2K. Eq. S8 allows rearranging the A_X / A_R and A_X / A_G expressions (Eqs. 4 and S6) as a function of $\rho_{L:DNA}$ to be compared with the experimental data. Substituting N_{Lf} in Eq. S6 with Eq. S8, and recalling the $f_{LPNP} = 1$ assumption, results in:

$$\frac{A_X}{A_R} = \frac{n}{1 + \left(\rho_{L:DNA.stab} / \rho_{L:DNA} \right) \cdot n \cdot (n-1)} \tag{S9a}$$

and

$$\frac{A_X}{A_G} = n \cdot \frac{\rho_{L:DNA.stab}}{\rho_{L:DNA}} \tag{S9b}$$

For $n = 1$, these expressions reduce to $A_X / A_G = 0.63 / \rho_{L:DNA}$ and $A_X / A_R = 1$. As can be seen in Fig. 2I, the agreement with the data is remarkable for A_X / A_G, especially taking into consideration that there are no fitting parameters. However, for A_X / A_R the discrepancy between the model and the data becomes more noticeable at larger $\rho_{L:DNA}$, even though the $A_X / A_R = 1$ line is still within the error bars.

The increasing discrepancy of the $A_X / A_R = 1$ line with the data suggests that, increasing the number of free liposomes, increases the number of collisions with LPNPs, with some of these collisions resulting in an LPNP with an extra liposome. If p is the probability of a free liposome enveloping an existing LPNP, the average number of liposomes per LPNP, n, can be estimated by:

$$n = 1 + p \cdot N_{Lf} / N_{LPNP} \tag{S10}$$

Combining Eqs. S8, S9 and S10, results in:

$$\frac{A_X}{A_R} = \frac{\left(\rho_{L:DNA.stab} + p \cdot \rho_{L:DNA} \right) \cdot (p+1) \cdot \rho_{L:DNA}}{\rho_{L:DNA.stab} \cdot \left(\rho_{L:DNA} + p \cdot \left(3 \cdot \rho_{L:DNA} - \rho_{L:DNA.stab} \right) \right) + \left(p \cdot \rho_{L:DNA} \right)^2} \tag{S11a}$$

and

$$\frac{A_X}{A_G} = \frac{\rho_{L:DNA.stab} + p \cdot \rho_{L:DNA}}{(p+1) \cdot \rho_{L:DNA}} \tag{S11b}$$

These modified expressions for A_X / A_R and A_X / A_G account for an increasing n when N_{Lf} increases and can be readily fitted to the data with p as the only fitting parameter. (Recall that here, for $\rho_{P:DNA} = 1.5$, $\rho_{L:DNA.stab}$ is fixed at 0.63, as determined in Fig. 2K). Note also that Eq. S11 reduces back to $A_X / A_G = 0.63 / \rho_{L:DNA}$ and $A_X / A_R = 1$ if p is zero ($n = 1$).

Figure 2I shows the best fit of Eq. S11 to the experimental data of LPNPs with 10 mol% PEG liposomes. Fig. S3 shows the best fits for the 5 and 0 mol% PEG systems. Unless otherwise noted, the fit is performed by a least-squares minimization of both A_X / A_R and A_X / A_G expressions simultaneously (Eqs. S11a and S11b). This way, each fit produces a single p value for each LPNP system that is a compromise for both A_X / A_R and A_X / A_G data. For comparison the results with the simple $1{:}1$ stoichiometry model are also plotted (dashed lines).

As discussed in the main text, for the 10 mol% PEG system, the modified $1{:}n$ model improves the fit to the A_X / A_R data, but not to A_X / A_G, which worsens. If the A_X / A_G data is fit alone, one obtains $p = 0$, which is the value expected for the $1{:}1$ stoichiometry model. Regarding the 5 and 0 mol% PEG systems, the $1{:}n$ model improves the fit for both A_X / A_R and A_X / A_G data, although if the A_X / A_G data is fit alone, the values of p are also lower (Table S6). Overall, this shows that the $1{:}1$ stoichiometry model is reasonable as a first order approximation and explains the data fairly well. The deviations observed when the number of free liposomes coexisting with LPNPs becomes significant are partly addressed, although not entirely, by the $1{:}n$ model described above.

Regarding the fitting parameters, we observe an increase of p as the degree of PEGylation in the liposomes decreases (this is even more noticeable if the A_X / A_G data is fitted alone – Table S6), indicating that liposomes with lower amounts of PEG are more likely to envelop an existing LPNP.

This observation agrees with the general understanding that PEGylated particles repel each other more strongly, hence lowering p.

S4. Determining the overlap between the green and red excitation volumes using single- and double-labelled liposomes

The measured cross-correlation amplitude ($A_{X.meas.}$) is limited by the amount of overlap between the green and red excitation volumes. We define the overlap volume correction factor (V_{crct}) as the factor that corrects the value of $A_{X.meas.}$ to the value of cross-correlation amplitude (A_X) that would be expected if the overlap between the two excitation volumes was perfect, according to Eq. S12,

$$A_X = A_{X.meas.} / V_{crct} \tag{S12}$$

To determine V_{crct} and correct the cross-correlation we performed a series of measurements with a set of samples consisting of a mixture between three PEGylated liposomes. Two of the liposomes were labelled with just one dye, one liposome type (L1) with 0.1 mol% of Atto-488 (green), and the other (L2) with 0.1 mol% Texas-red. The third liposome type (L3) was labelled with both dyes simultaneously. By gradually replacing liposomes L1 and L2, which are always in equal amounts and provide a non-colocalized signal, by liposomes L3, in which the two fluorescent probes are expected to be perfectly colocalized, V_{crct} can be determined. A related calibration approach was described recently by Werner et al[2].

The results presented in Fig. S4 show that both the $A_{X.meas.}/A_R$ and $A_{X.meas.}/A_G$ ratios increase as the single-labelled liposomes are gradually replaced by liposomes labelled with the two dyes. The maximum cross-correlation percentages detected in the sample with only dual-labelled liposomes were below 80%, due to the non-perfect overlap between the green and red confocal volumes. On the other hand, when the fraction of double-labelled liposomes is zero, there is still some amount of cross-correlation measured, due to the existence of some crosstalk between the dyes. (Note: as an exception to the remaining figures, where cross-correlation amplitudes are always corrected for crosstalk according to the procedure described in Bacia *et al*[3], the data in Fig. S4 is not corrected for crosstalk). The expected amplitudes for the auto- and cross-correlation functions in this setting are as follows:

$$A_G = \frac{\eta_{L1,G}^2 \cdot N_{L1} + \eta_{L3,G}^2 \cdot N_{L3}}{\left(\eta_{L1,G} \cdot N_{L1} + \eta_{L3,G} \cdot N_{L3}\right)^2} \tag{S13a}$$

$$A_R = \frac{\kappa_{Gr}^2 \cdot \eta_{L1,G}^2 \cdot N_{L1} + \eta_{L2,R}^2 \cdot N_{L2} + \left(\kappa_{Gr} \cdot \eta_{L3,G} + \eta_{L3,R}\right)^2 \cdot N_{L3}}{\left(\kappa_{Gr} \cdot \eta_{L1,G} \cdot N_{L1} + \eta_{L2,R} \cdot N_{L2} + \left(\kappa_{Gr} \cdot \eta_{L3,G} + \eta_{L3,R}\right) \cdot N_{L3}\right)^2} \tag{S13b}$$

$$A_{X.meas.} = \frac{V_{crct} \cdot \left(\kappa_{Gr} \cdot \eta_{L1,G}^2 \cdot N_{L1} + \eta_{L3,G} \cdot \left(\kappa_{Gr} \cdot \eta_{L3,G} + \eta_{L3,R}\right) \cdot N_{L3}\right)}{\left(\eta_{L1,G} \cdot N_{L1} + \eta_{L3,G} \cdot N_{L3}\right) \cdot \left(\kappa_{Gr} \cdot \eta_{L1,G} \cdot N_{L1} + \eta_{L2,R} \cdot N_{L2} + \left(\kappa_{Gr} \cdot \eta_{L3,G} + \eta_{L3,R}\right) \cdot N_{L3}\right)} \tag{S13c}$$

where κ_{Gr} is a factor describing the fraction of signal from the green liposomes (L1) that is detected in the red detector due to crosstalk. Both A_X/A_R and A_X/A_G values are fitted simultaneously with Eq. S13, allowing a robust determination of $V_{crct} = 0.746$.

Besides providing the correction factor for the volume overlap, this experiment also validates the suitability of FCCS to determine the colocalization of soft nanoparticles of ca. 100 nm.

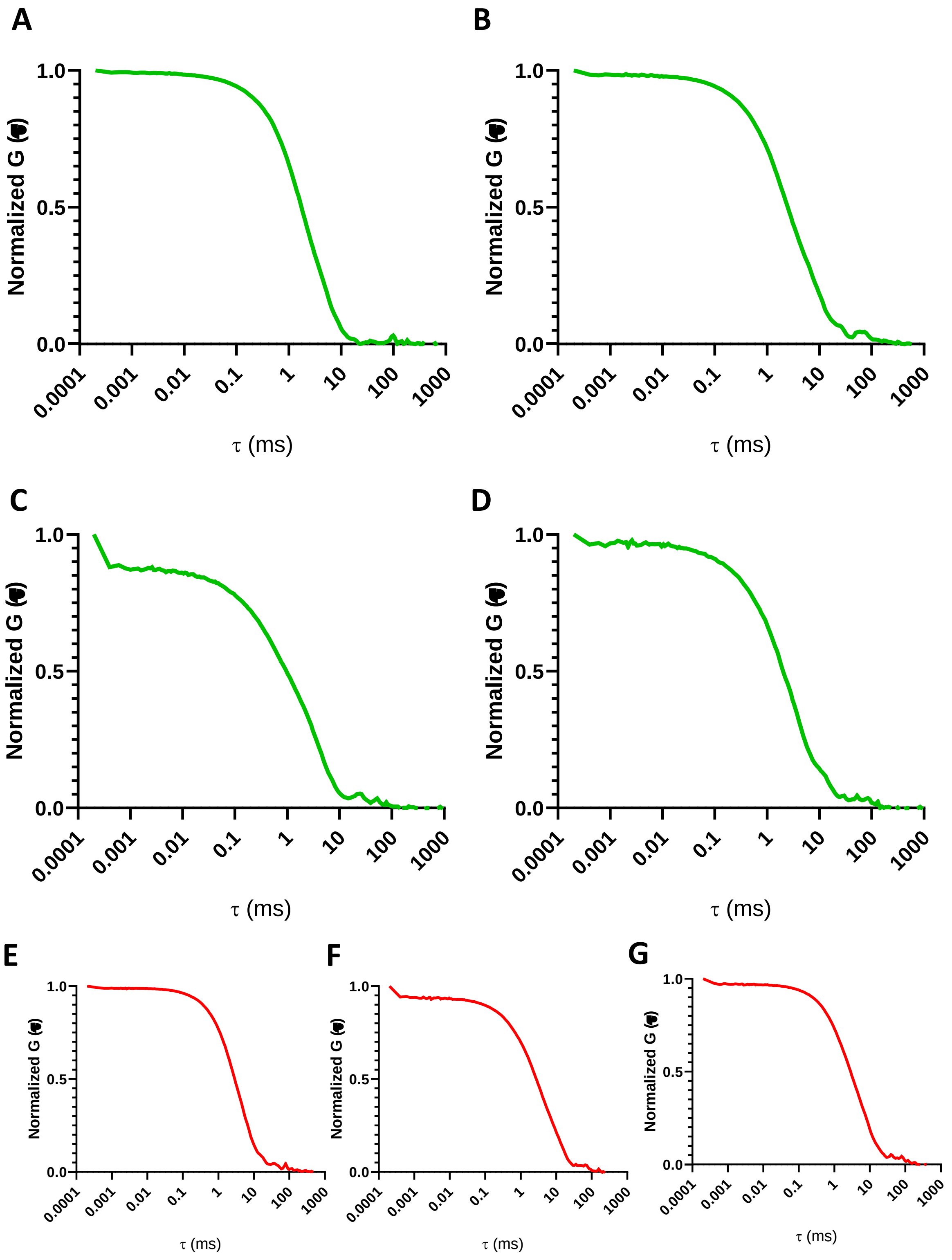

Fig. S1. Representative auto-correlation curves measured for each of the polyplex and liposome formulations. (**A-C**) Polyplex cores with Atto 488-labeled polylysine: (A) $\rho_{P:DNA} = 1$, (B) $\rho_{P:DNA} = 1.5$ and (C) $\rho_{P:DNA} = 3$). (**D**) Polyplex $\rho_{P:DNA} = 3$, where the DNA is labelled with YOYO-1. (**E-G**) Cationic liposomes labelled with Texas Red: (E) 0 mol% PEG, (F) 5 mol% PEG, (G) 10 mol% PEG. Green auto-correlation curves are shown in green and red auto-correlation curves are shown in red.

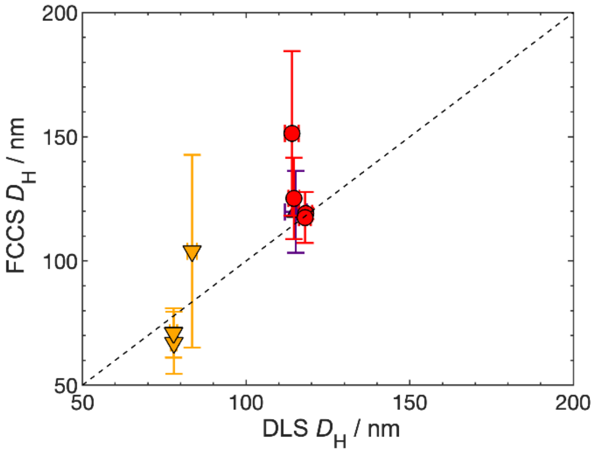

Fig. S2. Comparison between the LPNP hydrodynamic diameter (D_H) obtained with DLS and FCCS. The differences between sizes determined with DLS and FCCS are relatively small, indicating relatively low sample polydispersity[1].

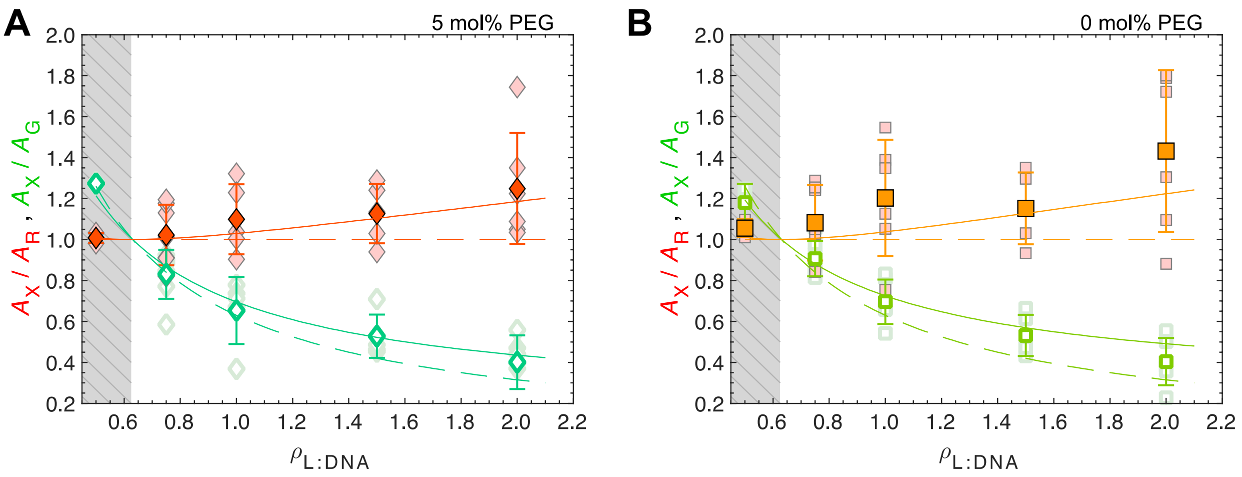

Fig. S3. 1:*n* polyplex:liposome stoichiometry model fits for the 5 and 0 mol% PEG LPNP systems. The 5 mol% PEG system is shown in (**A**). The 0 mol% PEG system is shown in (**B**). Individual measurements are represented by light-coloured symbols, while their means and respective error bars are represented in darker colours. The 1:1 and 1:n model fits are shown as dashed and straight lines, respectively, and as green-type and red-type colors for $^{A}X/A_{G}$ and $^{A}X/A_{R}$, respectively.

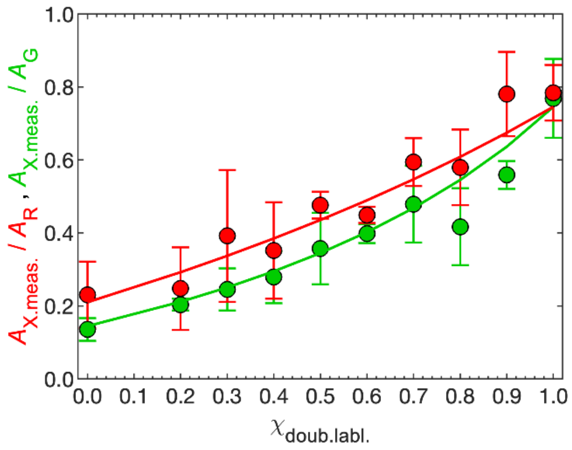

Fig. S4. Determination of the confocal overlap volume V_{crct}. By employing a mixture of liposomes labelled with either Atto-488 or Texas Red, and liposomes labelled with both dyes, the fraction of colocalization in solution can be controlled and compared with the measured cross-correlation. As the fraction of dual-labelled liposomes increases, $A_{X.meas.}/A_G$ (green symbols) and $A_{X.meas.}/A_R$ (red symbols) also increase, as expected. The estimated V_{crct}, obtained by fitting both curves simultaneously, is 74.6%. The measurements also confirm the suitability of FCCS to measure colocalization in soft self-assembled nanostructures of ca. 100 nm.

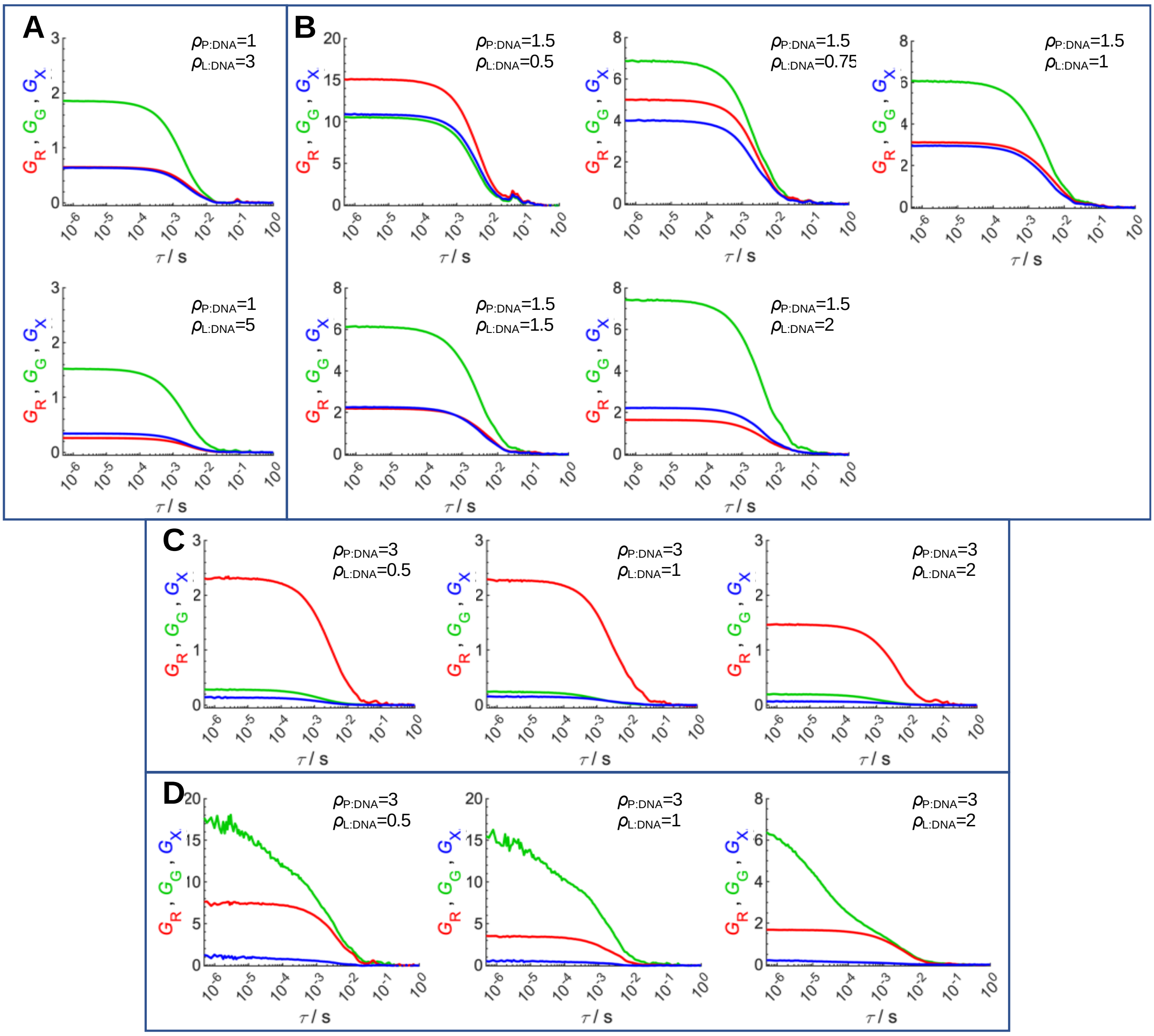

Fig. S5. Representative auto- and cross-correlation curves measured for each of the 10 mol% PEG LPNP formulations. The liposomes are labelled with Texas Red. (**A-C**) LPNPs using polyplex cores with Atto 488-labeled polylysine ($\rho_{P:DNA}=1$, 1.5 and 3). (**D**) LPNP formulations prepared employing a cationic polyplex core $\rho_{P:DNA}=3$, where the DNA is labelled with YOYO-1. Green auto-correlation curves are shown in green, red auto-correlation curves are shown in red, and cross-correlation curves are shown in blue. The existence of labelled free polylysine in excess in the $\rho_{P:DNA}=3$ system lowers dramatically the amplitude of the green autocorrelation to values where it becomes inaccurate. This hinders the determination of the association between cationic polyplexes and cationic liposomes (C). Hence, in this case ($\rho_{P:DNA}=3$), DNA was also labelled with YOYO-1 (D). The very low values of cross-correlation amplitude compared to both the autocorrelation amplitudes shows that cationic polyplexes and cationic liposomes do not associate.

Table S1. Characterization by FCS, DLS and electrophoretic mobility ($\zeta_{Potential}$) of polyplexes prepared with different charge ratios. Data are Means ± SD (N=3).

$\rho_{P:DNA}$	D_H (FCS) / nm	D_H (Z Average, DLS) / nm	PDI (DLS)	ζ Potential /mV
1	72.21 ± 2.94	120.7 ± 2.2	0.37 ± 0.02	-69 ± 3.5
1.5	106.59 ± 1.63	133.8 ± 3.4	0.29 ± 0.01	-36 ± 2.7
3	55.08 ± 5.82	79 ± 3.1	0.32 ± 0.03	+38.9 ± 1.8

Table S2. Characterization by FCS, DLS and electrophoretic mobility ($\zeta_{Potential}$) of cationic liposomes prepared with different PEGylation degrees. Data are Means ± SD (N=3).

PEG mol%	D_H (FCS) / nm	D_H (Z Average, DLS) / nm	PDI (DLS)	ζ Potential /mV
10	92.54 ± 1.73	99.2 ± 2.4	0.46 ± 0.034	+128.6 ± 2
5	80.79 ± 4.45	89.1 ± 5.8	0.44 ± 0.051	+120.6 ± 5.5
0	85.17 ± 1.67	91.9 ± 2.9	0.41 ± 0.044	+62.3 ± 1.5

Table S3. Characterization by DLS and electrophoretic mobility ($\zeta_{Potential}$) of LPNP formulations prepared employing different polyplexes ($\rho_{P:DNA}$=1, 1.5 and 3) combined with 10 mol% cationic liposomes. Data are Means ± SD (N=3).

$\rho_{P:DNA}$	$\rho_{L:DNA}$	D_H (Z Average) / nm	PDI	ζ Potential /mV
1	0.5	12184 ± 3269	17.71 ± 7.14	+17.8 ± 0.7
1	1	782.4 ± 336.5	1.487 ± 0.40	+35.9 ± 2.5
1	2	686.8 ± 19.2	2.46 ± 0.02	+48 ± 2.9
1	3	341.1 ± 143.8	1.003 ± 0.687	+62.4 ± 3
1	5	115.2 ± 3.3	0.377 ± 0.01	+65 ± 3.5
1.5	0.5	331.6 ± 372.77	0.8425 ± 1.327	+21.9 ± 5.7
1.5	0.75	118.2 ± 2.0	0.229 ± 0.02	+30.0 ± 8.6
1.5	1	114.6 ± 1.7	0.209 ± 0.037	+40.6 ± 1.8
1.5	1.5	114 ± 2.1	0.228 ± 0.009	+49.4 ± 0.4
1.5	2	118 ± 1.8	0.3 ± 0.043	+57 ± 0.8
3	0.5	77.9 ± 0.8	0.26 ± 0.03	+50.2 ± 0.7
3	1	77.8 ± 1.1	0.27 ± 0.03	+51.6 ± 2.7
3	2	83.5 ± 1.5	0.33 ± 0.03	+53.3 ± 0.1

Table S4. Size characterization by FCCS of LPNP formulations prepared employing different polyplexes ($\rho_{P:DNA}$=1, 1.5 and 3) combined with cationic liposomes (10% PEG). Data are Means ± SD (N=3).

$\rho_{P:DNA}$	$\rho_{L:DNA}$	Diffusion coefficient / $\mu m^2\ s^{-1}$	D_H (FCCS) / nm
1	5	4.15 ± 0.55	119.79 ± 16.53
1.5	0.5	3.37 ± 0.66	149.31 ± 26.64
1.5	0.75	4.98 ± 1.70	119.13 ± 2.34
1.5	1	3.97 ± 0.50	125.19 ± 16.33
1.5	1.5	3.34 ± 0.66	151.35 ± 33.13
1.5	2	4.20 ± 0.35	117.49 ± 10.21
3	0.5	7.51 ± 1.41	67.01 ± 12.53
3	1	7.00 ± 0.92	71.06 ± 9.93
3	2	5.16 ± 1.75	103.94 ± 38.83

Table S5. Summary of FCCS data for LPNPs. Results shown are obtained after fitting the auto- and cross-correlation curves with Eqs. 3 and 4. Data are Means ± SD (N≥3).

$\rho_{P:DNA}$	$\rho_{L:DNA}$	A_G	A_R	A_X	A_G^{-1} [a]	A_R^{-1} [a]	A_X / A_R [b]	A_X / A_G [b]
				**0 mol% PEG**				
1.5	0.5	8.97 ± 0.62	10.01 ± 0.50	10.55 ± 0.29	0.112 ± 0.008	0.100 ± 0.005	1.06 ± 0.04	1.18 ± 0.09
1.5	0.75	5.9 ± 2.3	4.78 ± 0.88	5.3 ± 1.7	0.191 ± 0.069	0.216 ± 0.044	1.08 ± 0.18	0.907 ± 0.086
1.5	1	7.9 ± 2.8	4.38 ± 0.81	5.2 ± 1.3	0.150 ± 0.083	0.235 ± 0.041	1.20 ± 0.28	0.70 ± 0.11
1.5	1.5	5.9 ± 1.3	2.74 ± 0.72	3.10 ± 0.66	0.177 ± 0.050	0.383 ± 0.084	1.15 ± 0.18	0.53 ± 0.10
1.5	2	7.0 ± 2.0	1.93 ± 0.40	2.67 ± 0.55	0.156 ± 0.058	0.54 ± 0.12	1.43 ± 0.39	0.40 ± 0.12
				**5 mol% PEG**				
1.5	0.5	10.43 ± 0.56	13.2 ± 1.0	13.28 ± 0.77	0.096 ± 0.005	0.076 ± 0.006	1.01 ± 0.03	1.27 ± 0.01
1.5	0.75	6.4 ± 2.9	5.0 ± 1.7	5.1 ± 1.7	0.185 ± 0.079	0.220 ± 0.072	1.02 ± 0.15	0.83 ± 0.12
1.5	1	8.0 ± 3.1	4.48 ± 0.84	4.82 ± 0.46	0.137 ± 0.037	0.231 ± 0.048	1.10 ± 0.17	0.65 ± 0.16
1.5	1.5	6.0 ± 1.8	2.9 ± 1.3	3.2 ± 1.1	0.186 ± 0.083	0.42 ± 0.24	1.13 ± 0.14	0.53 ± 0.11
1.5	2	6.8 ± 3.1	1.99 ± 0.75	2.44 ± 0.81	0.19 ± 0.13	0.57 ± 0.22	1.25 ± 0.27	0.40 ± 0.13
				**10 mol% PEG**				
1	3	1.81 ± 0.09	0.52 ± 0.13	0.652 ± 0.086	0.553 ± 0.027	1.98 ± 0.45	1.27 ± 0.16	0.360 ± 0.038
1	5	1.68 ± 0.14	0.32 ± 0.09	0.419 ± 0.035	0.598 ± 0.053	3.26 ± 0.82	1.35 ± 0.26	0.250 ± 0.013
1.5	0.5	10.4 ± 1.5	14.9 ± 1.8	14.3 ± 1.6	0.097 ± 0.015	0.068 ± 0.009	0.962 ± 0.044	1.37 ± 0.05
1.5	0.75	7.3 ± 4.3	5.2 ± 1.5	5.2 ± 2.1	0.171 ± 0.078	0.207 ± 0.055	0.98 ± 0.15	0.75 ± 0.10
1.5	1	7.1 ± 3.4	3.46 ± 0.58	3.7 ± 1.1	0.19 ± 0.14	0.295 ± 0.043	1.09 ± 0.32	0.58 ± 0.12
1.5	1.5	7.9 ± 3.7	3.5 ± 3.0	3.6 ± 2.2	0.147 ± 0.062	0.40 ± 0.16	1.19 ± 0.30	0.447 ± 0.077
1.5	2	6.5 ± 1.4	1.46 ± 0.19	2.02 ± 0.62	0.161 ± 0.040	0.696 ± 0.093	1.42 ± 0.48	0.314 ± 0.080
3	0.5	7.3 ± 5.4	5.6 ± 2.2	0.43 ± 0.19	0.31 ± 0.37	0.199 ± 0.073	0.076 ± 0.019	0.088 ± 0.062
3	1	7.8 ± 5.4	3.00 ± 0.58	0.24 ± 0.13	0.28 ± 0.33	0.342 ± 0.061	0.079 ± 0.044	0.039 ± 0.018
3	2	2.04 ± 0.14	1.73 ± 0.51	0.105 ± 0.019	0.492 ± 0.034	0.62 ± 0.19	0.062 ± 0.009	0.052 ± 0.012

[a]Note that for the simplified model of one liposome complexing with one polyplex (1:1 stoichiometry), A_G^{-1} and A_R^{-1} correspond to the total number of polyplexes ($N_{PPf} + N_{LPP}$) and liposomes ($N_{Lf} + N_{LPP}$) per confocal volume in the sample, respectively. [b]Note that within the same 1:1 stoichiometry approximation A_X/A_R and A_X/A_G indicate the fraction of polyplexes converted to LPNPs (f_{LPNP}) and fraction of liposomes used in LPNPs, respectively.

Table S6. Results for the probability of a free liposome enveloping an existing LPNP (p), as obtained through fitting Eqs. S11a and S11b to the A_X/A_R and A_X/A_G data. The second column shows the results obtained by fitting both A_X/A_R and A_X/A_G data simultaneously. This is the default method, whose results are shown in Figs. 2I and S3. The third column shows the results obtained by fitting solely A_X/A_G.

	A_X / A_G and A_X / A_R simultaneous fit	A_X / A_G fit
PEG mol%	*p*	*p*
0	0.349	0.213
5	0.214	0.137
10	0.190	0.000

References

(1) Gómez-Varela, A. I.; Gaspar, R.; Miranda, A.; Assis, J. L.; Valverde, R. R. H. F.; Einicker-Lamas, M.; Silva, B. F. B.; De Beule, P. A. A. Fluorescence Cross-Correlation Spectroscopy as a Valuable Tool to Characterize Cationic Liposome-DNA Nanoparticle Assembly. *Journal of Biophotonics* **2021**, *14* (1), e202000200–e202000200. https://doi.org/10.1002/jbio.202000200.

(2) Werner, S.; Ebenhan, J.; Haupt, C.; Bacia, K. A Quantitative and Reliable Calibration Standard for Dual-Color Fluorescence Cross-Correlation Spectroscopy. *ChemPhysChem* **2018**, *19* (24), 3436–3444. https://doi.org/10.1002/cphc.201800576.

(3) Bacia, K.; Petrášek, Z.; Schwille, P. Correcting for Spectral Cross-Talk in Dual-Color Fluorescence Cross-Correlation Spectroscopy. *ChemPhysChem* **2012**, *13* (5), 1221–1231. https://doi.org/10.1002/cphc.201100801.
